# Lactylation of PKM2 Suppresses Inflammatory Metabolic Adaptation in Pro-inflammatory Macrophages

**DOI:** 10.7150/ijbs.75434

**Published:** 2022-10-24

**Authors:** Jizhuang Wang, Peilang Yang, Tianyi Yu, Min Gao, Dan Liu, Jie Zhang, Chenghao Lu, Xuelian Chen, Xiong Zhang, Yan Liu

**Affiliations:** Department of burn, Shanghai Ruijin Hospital, Shanghai Jiaotong University School of Medicine, Shanghai, China.

**Keywords:** Lactylation, PKM2, Glycolysis, Macrophage

## Abstract

Emerging evidence suggests that metabolic adaptation is a vital hallmark and prerequisite for macrophage phenotype transition. Pyruvate kinase M2 (PKM2) is an essential molecular determinant of metabolic adaptions in pro-inflammatory macrophages. Post-translational modifications play a central role in the regulation of PKM2. However, doubt remains on whether lactylation in PKM2 exists and how lactylation modulates the function of PKM2. For the first time, our study reports that lactate inhibits the Warburg effect by activating PKM2, promoting the transition of pro-inflammatory macrophages towards a reparative phenotype. We identify PKM2 as a lactylation substrate and confirm that lactylation occurs mainly at the K62 site. We find that lactate increases the lactylation level of PKM2, which inhibits its tetramer-to-dimer transition, promoting its pyruvate kinase activity and reducing nuclear distribution. In short, our study reports a novel post-translational modification type in PKM2 and clarifies its potential role in regulating inflammatory metabolic adaptation in pro-inflammatory macrophages.

## Introduction

Macrophages are highly plastic immune cells that exhibit pleiotropic and coordinated responses to various microenvironmental signals 1-3. They present a complex phenotype that can vary from homeostatic, pro-inflammatory, and profibrotic to reparative phenotypes 4. During the natural wound-healing process, macrophages initially exhibit a pro-inflammatory phenotype, which is vital for killing microbes but leads to widespread damage to the surrounding tissues 5, 6. Soon thereafter, macrophages functionally transition towards a reparative phenotype 3, 7, that limits the inflammatory response and is responsible for tissue regeneration 8. Macrophages can respond to different microenvironmental signals and modulate their behavior along the inflammatory-reparative spectrum within an inflammatory response 3. For example, Di Zhang et al. report a time-dependent change in macrophage phenotype, with a decreased expression of the pro-inflammatory gene iNOS and increased expression of the tissue-repair gene ARG1 9. The research confirms that a macrophage cell can participate sequentially in both the induction and resolution of inflammation.

In recent years, the importance for wound healing of modulating macrophage responses along the inflammatory-reparative spectrum has become clear. Our previous work shows that halting the macrophages' transition from a pro-inflammatory phenotype to a reparative phenotype delays wound healing 10, 11. In addition, researchers have made numerous attempts to stimulate the polarization of macrophages toward a reparative phenotype for better wound healing outcomes 12, 13. However, the participating molecules and events that determine the phenotypic transition have not yet been fully elucidated.

Emerging evidence suggests that metabolic adaptation is a vital hallmark and prerequisite for macrophage phenotype transition 9, 14-16. It is well known that pro-inflammatory macrophages exhibit the Warburg effect. The Warburg effect means increased glycolysis and a weakened tricarboxylic acid (TCA) cycle; however, energy generation in reparative macrophages depends on the TCA cycle, fatty acids oxidation (FAO), and oxidative phosphorylation (OXPHOS) 17-19. Immunometabolism studies have shown that alterations in the metabolic profile of macrophages shape their activation state and function 20. Inhibition of glycolysis can stimulate peritoneal macrophages to transition from a pro-inflammatory phenotype to a reparative phenotype 19. The fact reveals that interfering with metabolic profiles in macrophages may be critical to regulating phenotype.

Many studies have highlighted the critical role of pyruvate kinase M2 (PKM2) in regulating the Warburg effect 21-24. There are four pyruvate kinase isoforms (M1, M2, L, and R) that exist in mammals, but the immune cells preferentially express the isoforms PKM1 and PKM2, which are generated by alternative splicing of the PKM gene 24. On the one hand, PKM1 is a tetrameric protein that is enzymatically active and can efficiently convert phosphoenolpyruvate to pyruvate. On the other hand, PKM2 is subject to complex, allosteric regulation that directs its pyruvate kinase activity and mainly exists in monomeric or dimeric forms, which are less enzymatically active than PKM1 23. Promoting PKM2 pyruvate kinase activity results in decreased lactate production, which is a phenomenon known as the PKM2 paradox in the Warburg effect. A study has shown that oxaloacetate accumulates and promotes the Warburg effect by promoting PKM2 pyruvate kinase activity 25. With the reduction in PKM2 activity, the monomeric and dimeric forms of PKM2 can translocate to the nucleus, interact with Hif-1α and regulate the expression of numerous pro-glycolytic enzymes 21. Nuclear dimeric PKM2 also functions as a histone kinase and upregulates c-Myc expression, promoting the expression of pro-glycolytic enzymes that induces the Warburg effect 26.

Post-translational modifications (PTMs) extensively regulate the function of proteins, shaping cell phenotype to accommodate the environment 9, 27. These modifications alter the structure and function of the target protein and play a key role in the endogenously allosteric regulation of PKM2 23, 28. Other studies have shown that various PTMs significantly regulate the pyruvate kinase activity of PKM2. For example, citrullination of PKM2 R106 reprograms cross-talk between PKM2 ligands to promote its activity 29, and SUMOylation of PKM2 K270 triggers conformation change from the tetrameric to the dimeric form of PKM2, reducing pyruvate kinase activity 30. In addition, PKM2 acetylation at lysine 433 promotes its activity, 31 while O-GlcNAcylation or K311 succinylation inhibits PKM2 catalytic activity and promotes the Warburg effect 32, 33. These studies show that investigating the PTMs of PKM2 helps enrich understanding of the regulatory mechanism of PKM2. A recent study identified a novel post-translational modification: lysine lactylation, which lactate can stimulate 9. Although it is well established that PKM2 directs lactate production, whether lactate and lactylation regulate PKM2 pyruvate kinase activity or function remains largely unknown. Therefore, our study aims to investigate the regulatory effect of lactate and lactylation on PKM2. We further study the potential role of lactate in the metabolic adaptation and phenotypic transition of pro-inflammatory macrophages. For the first time, we identify PKM2 as a lactylation substrate. We provide evidence that lactylation of PKM2 increases its pyruvate kinase activity, and reduces its tetramer-to-dimer transition and nuclear distribution. In addition, we show that lactate decreases glycolysis by activating PKM2, promoting the phenotypic transition of macrophages along the inflammatory-reparative spectrum.

## Materials and Methods

### Antibodies and reagents

L-(+)-lactic acid (L6402; lactate) was purchased from Sigma. PKM2 enzymatic inhibitor (Compound 3K, S8616) and PKM2 activator (DASA-58, S7928) were from Selleck. Lipopolysaccharide (LPS, tlrl-3pelps) was from InvivoGen. Mouse interleukin-4 (IL-4) protein (51084-MNAE) was from Sino biological Inc.; Millicell EZ Slide (PEZGS0816) was from Merck Millipore Ltd.

The antibodies anti-PKM2 (#4053S), anti-Arginase-1 (ARG1, #93668S), the HRP-linked secondary antibodies anti-rabbit IgG (#7074S), and anti-mouse IgG (#7076S) were from Cell Signaling Technologies. Anti-β-actin (#66009-1-Ig), anti-Flag (#20543-1-AP), and anti-PKM1 (#15821-1-AP) were from Proteintech. Anti-Histone H3 (#A2348) was from ABclonal. Anti-CD68 (#ab955) was from Abcam. Anti-L-Lactyl Lysine (#PTM-1401RM) was from PTM Bio Inc. Anti-Inducible nitric oxide synthase (iNOS; #PA1-036), anti-rabbit Alexa flour 568 (#A10042), and anti-mouse Alexa flour 488 (#A21202) were from Thermo Fisher. Primary and secondary antibodies were used at the concentrations recommended by the manufacturers.

### Cell culture

To isolate murine bone-marrow-derived macrophage cells (BMDM), bone marrow was separated from the 6-10-week-old C57BL/6 mice femurs. Then cells were resuspended at a concentration of 1 × 10^6^ cell/ml in a complete Dulbecco's modified eagle medium. Recombinant macrophage-colony stimulating factor was added to a final concentration of 20 ng/ml and 10 ml media were plated per 100 mm Petri dish. The start of culture was considered to be Day 0. On Days 3 and 5, the fresh culture medium containing M-CSF was changed to 20 ng/ml. On Day 6, the cells were considered naïve macrophages and ready for experimentation. The 293T cells were from the Type Culture Collection of the Chinese Academy of Sciences (Shanghai, China). Cells were grown in a DMEM medium supplemented with 10% fetal bovine serum and maintained in an incubator at 37 °C, 5% CO2, and 95% air. Overexpression of wild type (WT) or mutant PKM2 (flag-tagged) was introduced into 293T cells by transfection with plasmids synthesized at Shanghai Xitubio Biotechnology (Shanghai, China). Overexpression of mouse WT or K62R mutant PKM2 (flag-tagged) was introduced into BMDM cells by transfection with lentivirus synthesized at Shanghai Xitubio Biotechnology (Shanghai, China). Cell clones were selected with 0.5 μg/ml puromycin.

### Western blot analysis and PKM2 crosslinking

Western blot analysis (WB) was performed as previously described 34. Total protein was extracted and measured with the BCA assay. Proteins (20 μg) were separated by ExpressPlus™ PAGE Gel (GenScript, M42015C) and then transferred to a nitrocellulose membrane (Millipore, MA, USA). The membrane was blocked with 5% skimmed milk and incubated overnight at 4 °C with indicated primary antibodies. The membranes were then incubated with corresponding secondary antibodies and visualized using enhanced chemiluminescence. For PKM2 crosslinking experiments, cells were collected and washed three times with phosphate buffer saline (PBS, pH 8.0), then incubated in PBS (pH 8.0) and 500μM disuccinimidyl suberate (DSS; Thermo Fisher; 21655) for 30 min at 37 °C. Cells were then washed and lysed in Laemmli Sample Buffer.

### Immunofluorescence

Cells cultured on EZ Slides were fixed in 4% paraformaldehyde for 15 min at room temperature. Paraffin-embedded wound tissue sections were heated at 60 °C overnight, dewaxed in xylene, rehydrated in a graded series of ethanol solutions, and treated with 0.01 mol/L citrate buffer (pH 6.0) for antigen retrieval. Then, the slides were treated with 0.5% TritonX-100 for 10 min and incubated with 5% bovine serum albumin for 1 hr at room temperature, then incubated with the appropriate primary antibody overnight at 4 °C. After washing, the molecules of interest were visualized after incubation with the secondary antibody. Images were obtained with a Carl Zeiss Axioskop 2 plus (Carl Zeiss) microscope using ZEN software (Carl Zeiss) and processed using ZEN software (Carl Zeiss). Negative control slides were stained with secondary antibodies only. A fixed threshold value was applied to exclude background intensity for each image.

### RNA isolation and real-time quantitative polymerase chain reaction

Total RNA was isolated from cells using Trizol and cDNA was synthesized using the HiScript® III-RT SuperMix kit (Vazyme, R323-01). The cDNA samples were subjected to a real-time quantitative polymerase chain reaction (RT-qPCR) using the ChamQ Universal SYBR qPCR Master Mix (Vazyme, Q711-02) performed on an ABI 7500 Sequence Detection System (Thermo Fisher). All procedures were performed according to the manufacturer's protocols. The primer sequences are listed in Table 1.

### Nuclear and cytoplasmic protein extraction

For experiments requiring nuclear and cytoplasmic protein extraction, nuclear and cytoplasmic extracts were isolated from cells using the Nuclear and Cytoplasmic Protein Extraction Kit (Beyotime, P0028), following the manufacturer's instructions.

### Pyruvate kinase activity

Cells were lysed, and the supernatant was then used in the enzyme assays to test pyruvate kinase activity, following the manufacturer's instructions (Nanjing Jiancheng Bioengineering Institute, A076-2-1). Pyruvate kinase activity was normalized to protein concentration.

### Immunoprecipitation

Immunoprecipitation (IP) was performed according to the manufacturer's instructions for the Pierce™ Classic Magnetic IP/Co-IP Kit (Thermo Fisher, 88804). First, the corresponding primary antibody was incubated with cell lysate overnight at 4 °C to form the immune complex. Then, Pierce Protein A/G Magnetic Beads were incubated with the antigen sample and antibody mixture at room temperature for 1 hr with mixing. Finally, the bound protein was eluted for western blot analysis.

### Wounding Procedure

Male C57BL/6 mice (6-10-week-old) were obtained from the Shanghai Laboratory Animal Center and housed at the Animal Science Center of the Shanghai Jiao Tong University, School of Medicine (SJTUSM). All experimental procedures were performed per the Animal Care Committee of SJTUSM and were approved by the SJTUSM Institutional Animal Care and Use Committee (SJTUSM IACUC; B-2019-010).

Each mouse was anesthetized (intraperitoneal injection of 1% sodium pentobarbital, 60 mg/kg), and its hair was thoroughly removed. The wounds were made as previously described 35. Two 9-mm, full-thickness skin wounds were made on the back. The wounds of mice in the control group (CTRL) were treated with saline. In contrast, the wounds of mice in the lactate (LA) or lactate + PKM2 enzymatic inhibitor groups (LA+Pi) were topically administered with 20 mM lactate or 1.2 μM compound 3K at 3 days and 5 days post-injury. On Days 3, 5, 7, and 9 post-injury, the mice were anesthetized, the wounds were photographed, and wound closure was measured. All images were analyzed with ImageJ software. Wounds and an additional 5 mm of the surrounding normal skin tissues were collected on Days 5 and 12 post-injury. Paraffin sections of the wound tissues were stained with hematoxylin and eosin (H&E).

### Extracellular acidification and oxygen consumption rate

The extracellular acidification rates (ECAR, indicative of glycolysis) and oxygen-consumption rates (OCR, indicative of respiration) were monitored with an XF24 Extracellular Flux analyzer (Seahorse Biosciences, with Agilent Seahorse XF Glycolytic Rate Assay kit) and an XF96 Extracellular Flux analyzer (Seahorse Biosciences, with Agilent Seahorse XF Cell Mito Stress Test kit and Agilent Seahorse XF Glycolysis Stress Test kit). BMDMs were plated at 200,000 cells per well in XF24 plates overnight, then 100 ng/ml lipopolysaccharide (LPS) for 4 hr, followed by ±1.2 μM PKM2 enzymatic inhibitor for 20 hr. BMDMs were seeded into XF96 plates at 50,000 cells per well overnight then incubated with LPS (LPS), LPS + PKM2 enzymatic inhibitor (LPS+Pi), LPS + lactate (LPS+LA), or LPS + lactate + PKM2 enzymatic inhibitor (LPS+LA+Pi) for 24 hr. Detailed steps were followed according to the instructions provided by Seahorse Bioscience.

### IP-mass spectrometry

The mouse BMDM cells were incubated with 100 ng/ml LPS for 4 hr, followed by vehicle (LPS group) or 20 mM lactate (LPS-LA group) for 20 hr. Then, the anti-PKM2 antibody was used for IP. Immunoprecipitated proteins were subjected to electrophoresis by ExpressPlus™ PAGE Gel electrophoresis and brilliant Coomassie blue staining. Enriched proteins were further analyzed for lactylation identification by mass spectrometry.

### Statistical analysis

All results are represented as mean ± SEM. Parametric tests were used for data that conform to the normal distribution (Shapiro-Wilk test). Differences between the two groups were analyzed using a Student's t-test. Differences in multi groups were analyzed with a one-way analysis of variance (ANOVA) followed by Tukey's post-test. A value of *p*<0.05 was considered statistically significant. All statistical analysis was performed using the GraphPad Prism 9.0 (Aspire Software International).

## Results

### PKM2 regulates glycolysis in LPS-induced macrophages

**Figure 1:**
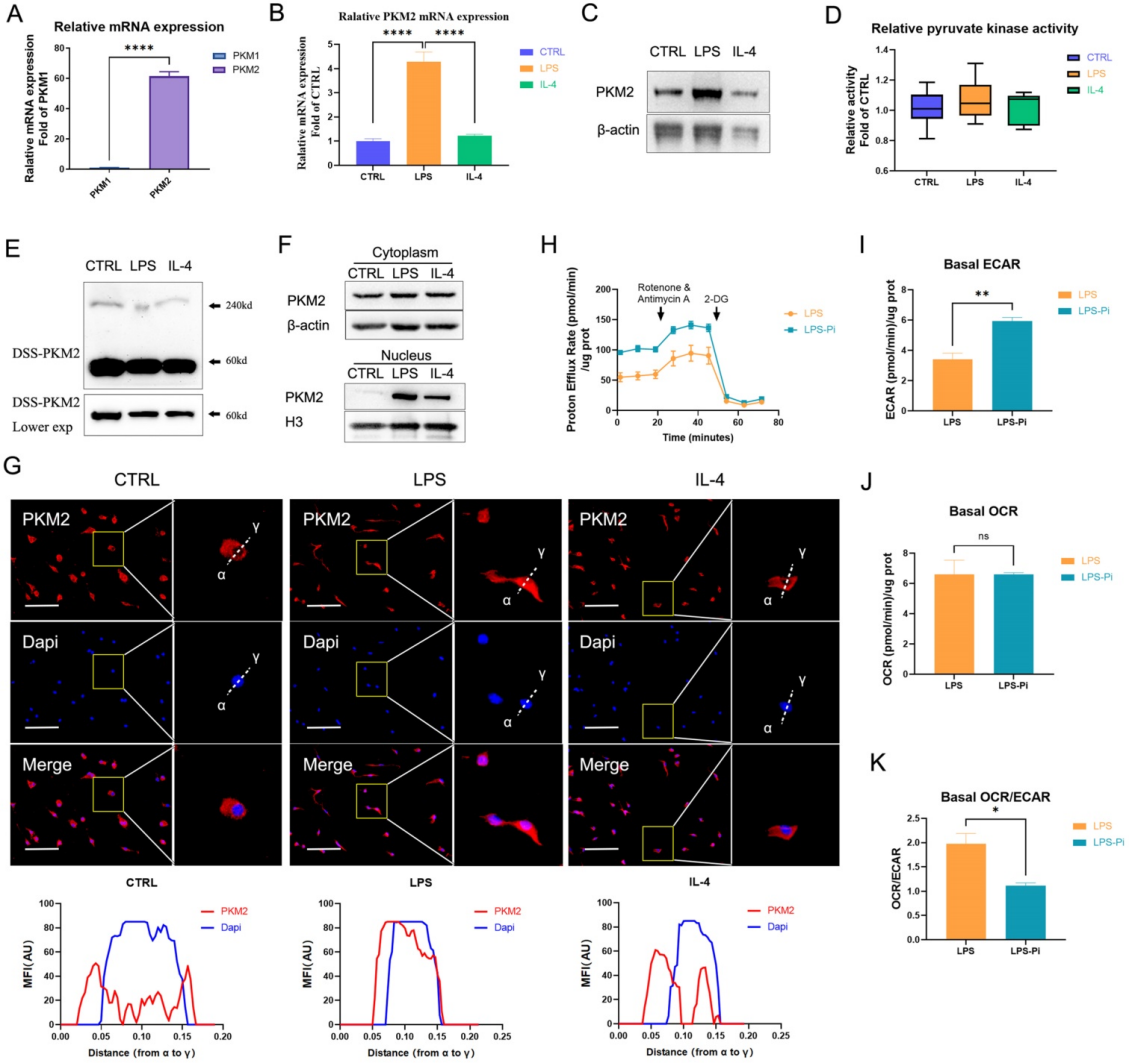
** PKM2 regulates glycolysis in lipopolysaccharide-induced macrophages. (A)** The mRNA from naïve bone-marrow-derived macrophage (BMDM) cells was extracted to test PKM1 and PKM2 expression (n = 3). **(B)** Naïve BMDM cells (CTRL) were incubated with 100 ng/ml lipopolysaccharide (LPS) or 25 ng/ml IL-4 (IL-4) for 24 hr, then mRNA was extracted to test PKM2 expressions, which were normalized to the β-actin mRNA levels (n=3). **(C)** BMDM cell proteins were analyzed by western blotting (WB) for the PKM2 level. **(D)** BMDM cell lysates were assayed for pyruvate kinase activity (n=6). **(E)** BMDM cells were collected and crosslinked with disuccinimidyl suberate (DSS). The tetrameric (240KD), dimeric (120KD), and monomeric (60KD) forms of PKM2 were analyzed by WB. **(F)** Cytosolic and nuclear proteins were purified from the BMDM cells separately for PKM2 level analysis. **(G)** The localization of PKM2 was analyzed by immunofluorescence staining. The nuclei were stained with 4',6-diamidino-2-phenylindole (DAPI; blue). The line charts represent fluorescence intensity (MFI), presenting the distance from α to γ. **(H)** Naïve BMDMs were incubated with 100ng/ml LPS (4 hr), followed by ±1.2 µM PKM2 enzymatic inhibitor (Pi) for 20 hr. Proton efflux rate (PER; reflecting glycolytic rate) in BMDM cells is shown. Basal extracellular acidification rates (ECAR; **I**), basal oxygen-consumption rate (OCR; **J**), and basal OCR/ECAR **(K)** in BMDM cells treated with LPS ± Pi are shown. Data represents mean ± SEM, scale bar = 100 µm. *p < 0.5, ** p < 0.01, **** p < 0.0001.

Immune cells preferentially express two pyruvate kinase isoforms PKM1 and PKM2 that are generated by alternative splicing of the PKM gene 24. The data showed that the expression of PKM2 mRNA in naïve BMDM cells was significantly higher than the expression of PKM1 (Figure 1A). Moreover, as shown in Figure S2, we detected the protein levels related to the expression of the two pyruvate kinase subtypes in different cells. The PKM2 level was dramatically higher than the PKM1 level in naïve BMDM cells. These facts confirmed that PKM2 was more abundantly expressed than PKM1 in BMDM cells. Consistent with previous findings, the PKM2 mRNA expression and protein level increased dramatically after 24 hr of 100 ng/ml LPS incubation, which was also markedly higher than in 25 ng/ml IL-4 induced macrophages (Figures 1B, C) 21. Interestingly, although the PKM2 protein level of LPS-induced macrophages was significantly higher than that of naïve and IL-4-induced macrophages, there was no significant difference in pyruvate kinase activity (Figure 1D). The DSS crosslinking of lysates from BMDM cells suggested that the level of the tetrameric form of PKM2 (240KD) in LPS-induced macrophages was significantly lower than that in naïve and IL-4-induced macrophages (Figure 1E). The dimeric/monomeric forms, in contrast to the tetrameric form of PKM2, can enter the nucleus so we assessed the subcellular localization of the PKM2. As depicted in Figure 1F, LPS caused an increased expression of PKM2 in the nucleus. Through immunofluorescence, we observed that the level of PKM2 in the nuclei of LPS-induced macrophages was significantly higher than that in naïve and IL-4-induced macrophages (Figure 1G). These results suggest that PKM2 is overexpressed in LPS-induced macrophages but is more inactive than in naïve and IL-4-induced macrophages.

Previous studies have revealed the critical role of PKM2 in regulating the Warburg effect 21-24. We measured the impact of reducing PKM2 pyruvate kinase activity on LPS-incubated BMDM cells' ECAR (representing glycolysis) and OCR (representing OXPHOS). The results demonstrate that a PKM2 enzymatic inhibitor (Pi; 1.2μM Compound 3K) caused a significant increase in the glycolytic rate (Figures 1H, I) but did not lead to a change in the OCR (Figure 1J) in LPS-incubated macrophages. The basal OCR: ECAR ratio revealed that reducing PKM2 pyruvate kinase activity induced a metabolic transition to higher glycolysis in LPS-incubated macrophages (Figure 1K). These results suggest that reducing PKM2 pyruvate kinase activity enhanced the Warburg effect, promoting inflammatory metabolic adaptation in LPS-incubated macrophages.

### Lactate activates PKM2 in LPS-induced macrophages

**Figure 2:**
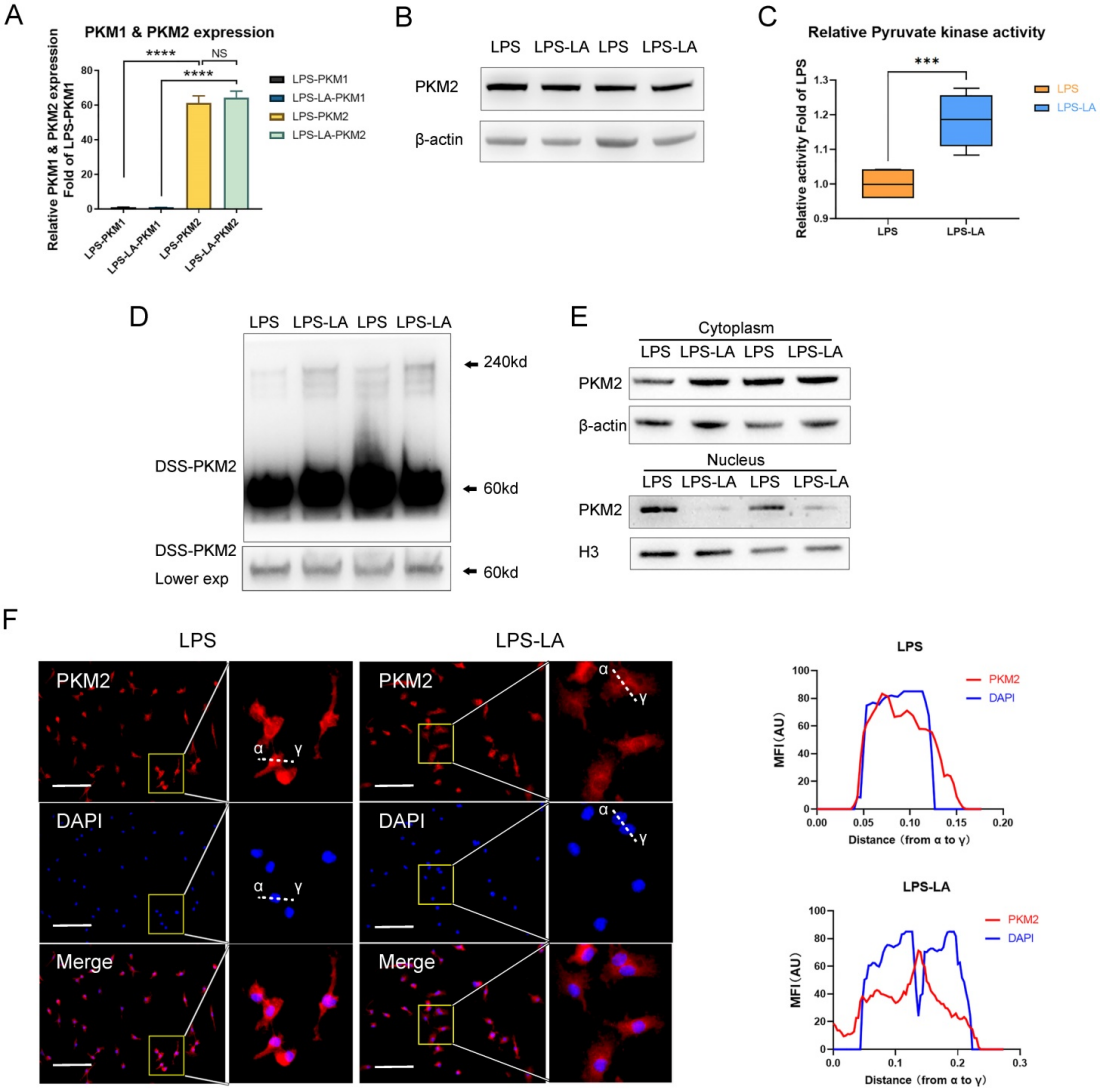
** Lactate activates PKM2 in lipopolysaccharide-induced macrophages. (A)** Naïve bone-marrow-derived macrophage (BMDM) cells were incubated with lipopolysaccharide (LPS) for 4 hr, followed by vehicle (LPS) or lactate (LPS-LA) for 20 hr. The mRNA was extracted for testing PKM1 and PKM2 expression (n=3). **(B)** BMDM cell proteins were analyzed by western blotting (WB) for the PKM2 level. **(C)** BMDM cell lysates were assayed for pyruvate kinase activity (n = 6). **(D)** BMDM cells were collected and crosslinked with disuccinimidyl suberate (DSS). The tetrameric (240KD), dimeric (120KD), and monomeric (60KD) forms of PKM2 were analyzed by WB. **(E)** Cytosolic and nuclear proteins were purified from the BMDM cells separately for PKM2 level analysis. **(F)** The localization of PKM2 was analyzed by immunofluorescence staining. The nuclei were stained with 4',6-diamidino-2-phenylindole (DAPI; blue). The line charts represent fluorescence intensity (MFI), presenting the distance from α to γ. WB data represents three independent experiments. Data represents mean ± SEM, Scale bar = 100 µm. ***p < 0.001, ****p < 0.0001.

PKM2 regulates glycolysis and lactate production 25; however, it remains unclear if lactate regulates PKM2. Therefore, we first examined the effect of lactate on PKM2 mRNA expression and protein level in BMDM cells. The results show that the intervention of exogenous lactate (20mM) changed neither the mRNA expression of PKM2 (Figure 2A) nor the PKM2 protein level (Figure 2B) in LPS-induced macrophages. Lactate significantly promoted the pyruvate kinase activity of LPS-induced macrophages (Figure 2C). The DSS crosslinking of lysates from BMDM cells suggests that the level of PKM2 tetrameric form (240KD) in the LPS-LA group was significantly higher than that in the LPS group (Figure 2D). What is more, the level of PKM2 in the nuclei of the LPS-LA group was considerably lower than that in the LPS group, which was confirmed by immunofluorescence (Figures 2E, F). These results suggest that lactate activates PKM2 in LPS-induced macrophages.

### Lactate inhibits glycolysis and promotes the transition of LPS-induced macrophages to a reparative phenotype by activating PKM2

**Figure 3:**
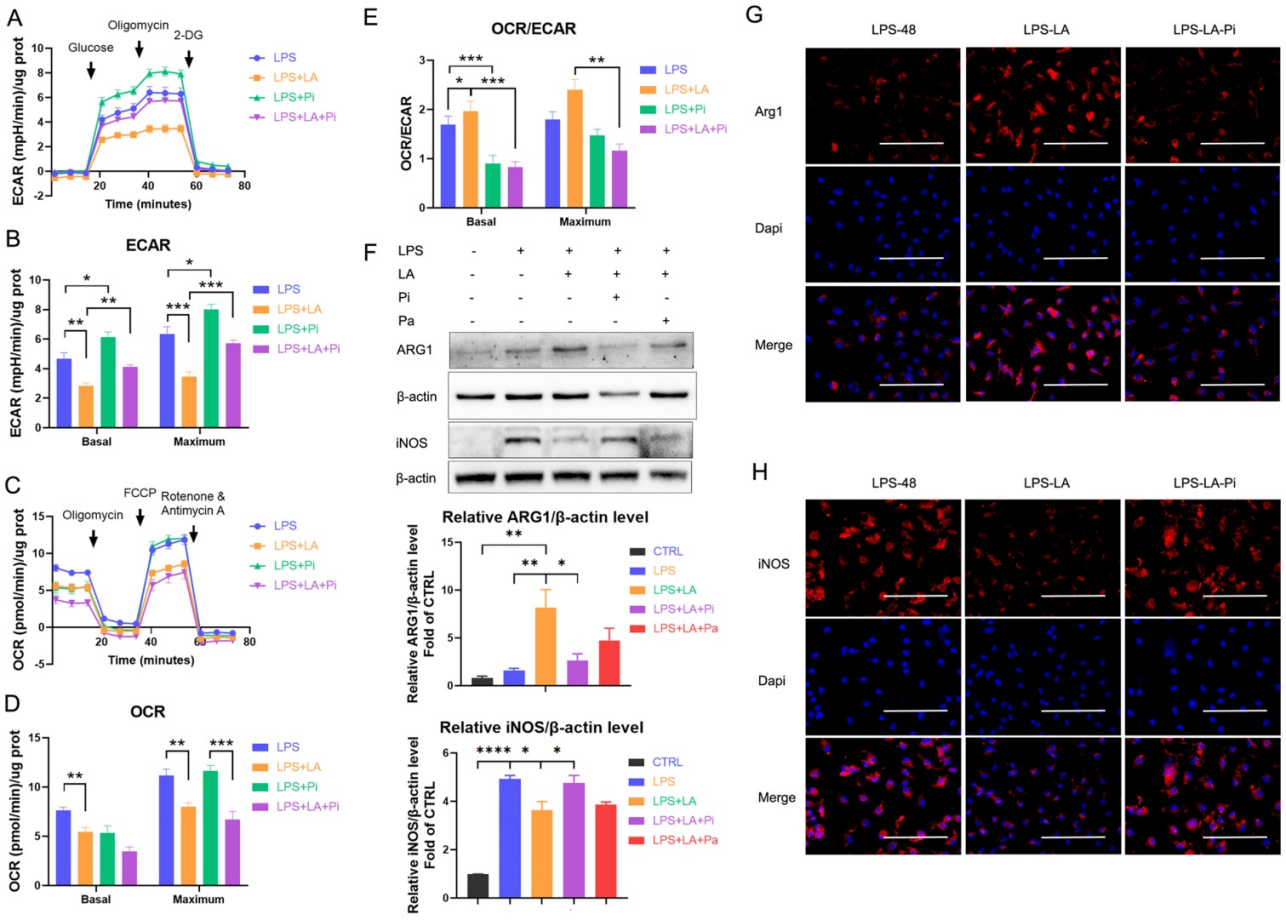
** Lactate inhibits glycolysis and promotes the transition of lipopolysaccharide-induced macrophages to a reparative phenotype by activating PKM2.** Naïve bone-marrow-derived macrophage (BMDM) cells were incubated with lipopolysaccharide (LPS), LPS + PKM2 enzymatic inhibitor (LPS+Pi), LPS + lactate (LPS+LA), or LPS + lactate + PKM2 enzymatic inhibitor (LPS+LA+Pi) for 24 h. Then the rate of extracellular acidification (ECAR; **A**), basal and maximum capacity of ECAR **(B)**, rate of oxygen consumption (OCR; **C**), basal and maximum capacity of OCR **(D)**, and OCR/ECAR **(E)** were analyzed by using a respirometry and metabolomics instrument Seahorse XF96 extracellular flux analyzer. **(F)** Naïve BMDM cells were treated with vehicle (CTRL), LPS (LPS-48), LPS + lactate (LPS-LA), LPS + lactate + PKM2 enzymatic inhibitor (LPS-LA-Pi) or LPS + lactate + PKM2 activator (LPS-LA-Pa) for 48 h. Then cell proteins were analyzed by western blotting for the iNOS and ARG1 levels. The blots are representative of three independent experiments. Representative images of immunofluorescence staining of cells was performed for iNOS **(G)** or ARG1 **(H)** levels. Data represents mean ± SEM, scale bar = 100 µm. *p < 0.5, **p < 0.01, ***p < 0.001.

Next, we measured the impact of lactate on LPS-induced macrophages' ECAR and OCR levels. As the data shows, lactate significantly reduced the ECAR level in the LPS-incubated macrophages (Figures 3A, B; LPS+LA group vs LPS group). In addition, we observed that lactate reduced the OCR level (Figures 3C, D). Overall, lactate resulted in a significant up-regulation of macrophages' OCR: ECAR ratio (Figure 3E). In contrast, the PKM2 enzymatic inhibitor significantly increased the ECAR level (Figures 3A, B; LPS+Pi group vs LPS group) but did not change the OCR level (Figures 3C, D), which decreased the OCR: ECAR ratio (Figure 3E). Significantly, the PKM2 enzymatic inhibitor reversed the effect of lactate. The LPS+LA+Pi group showed a higher ECAR level (Figures 3A, B) with a lower OCR: ECAR ratio (Figure 3E) than the LPS+LA group. These results suggest that lactate may impair inflammatory metabolic adaptation in LPS-induced macrophages by activating PKM2.

Di Zhang et al. recently reported a time-dependent change in macrophage phenotype associated with decreased expression of pro-inflammatory gene iNOS and increased expression of the tissue-repair gene ARG1 9. The research reveals that a macrophage cell can transit from a pro-inflammatory phenotype to a reparative phenotype. Recent studies suggest that inhibition of glycolysis or lactate can stimulate this transition in macrophages 19, 36. We analyzed whether lactate promotes the transition of LPS-induced macrophages to a reparative phenotype by activating PKM2. In BMDM cells, 48 hr after LPS incubation (100 ng/ml LPS), lactate increased the ARG1 level but decreased the iNOS level (Figures 3F-H; LPS-LA group vs LPS-48 group). The LPS-LA-Pi group showed a higher iNOS level and a lower ARG1 level than the LPS-LA group (Figures 3F-H). These results support the theory that lactate partially promotes the transition of LPS-induced macrophages to a reparative phenotype by activating PKM2.

### Lactate promotes the transition of wound macrophages to a reparative phenotype and accelerates wound healing in mice by activating PKM2

The transition of macrophages from a pro-inflammatory phenotype to a reparative phenotype plays a crucial role in wound healing 7, 10. Numerous studies have stimulated the polarization of macrophages toward a reparative phenotype for better wound healing outcomes 12, 13. In this study, we constructed a skin-wound model using mice to test whether lactate promotes the transition of wound macrophages from a pro-inflammatory phenotype to a reparative phenotype and accelerates wound healing by activating PKM2.

**Figure 4:**
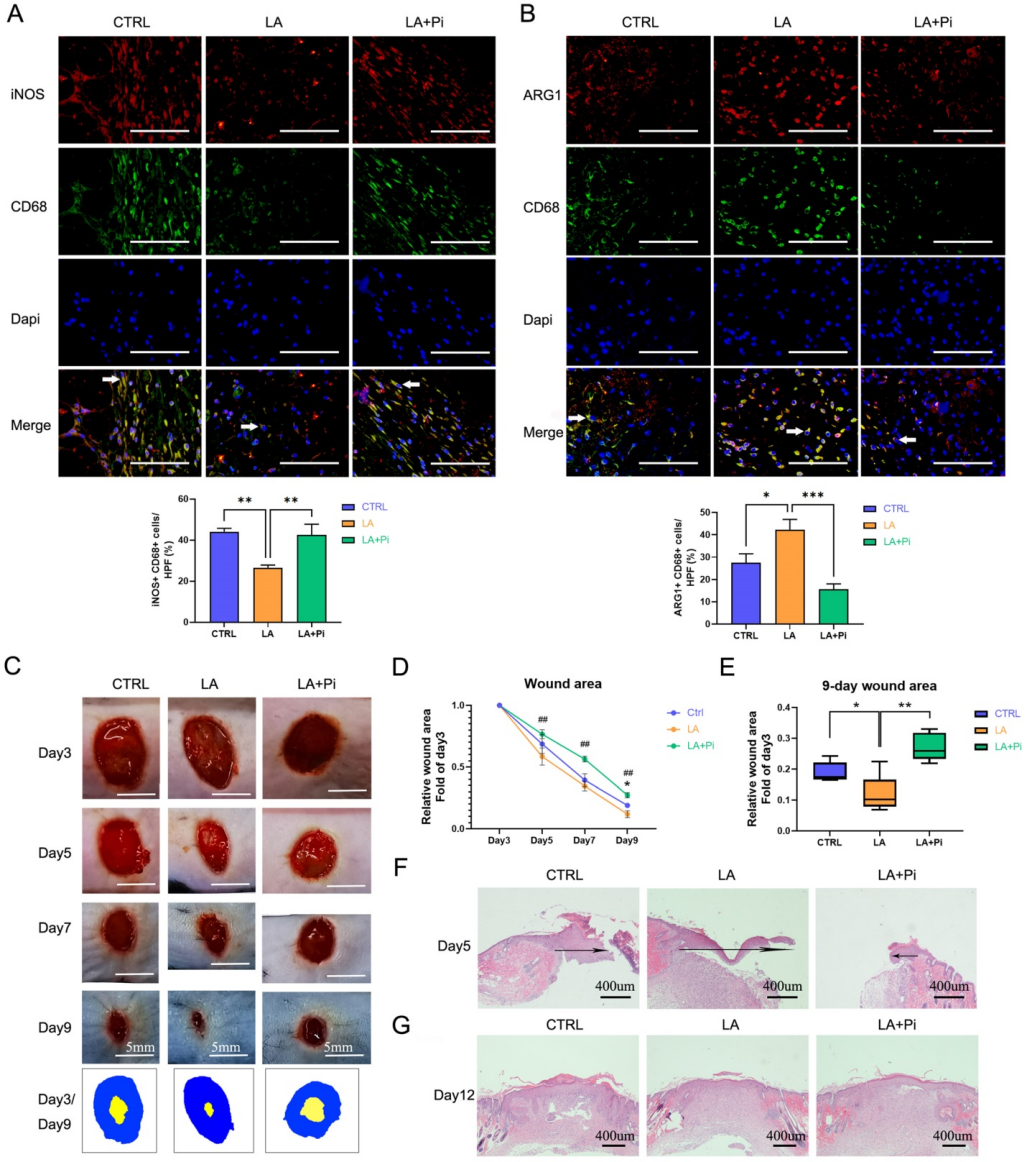
** Lactate promotes the transition of wound macrophages to a reparative phenotype and accelerates wound healing in mice by activating PKM2.** Eighteen mice were randomly divided into three intervention groups: the CTRL group (treated with vehicle, n = 6), the LA group (treated with 20 mM lactate, n = 6), and the LA+Pi group (treated with lactate + 1.2 µM PKM2 enzymatic inhibitor, n = 6). Immunofluorescence staining with iNOS + CD68 **(A)** or ARG1 + CD68 **(B)** was performed to analyze wound macrophages on Day 5 post-injury. Scale bar = 100 µm. n = 6. **(C)** The photographs of skin wounds and schematic diagram of comparison of wound area on Day 3 and Day 9. Scale bar = 5 mm. **(D)** Statistical results of relative wound area (fold of the wound on Day 3). # means the statistical difference between the LA and LA + Pi groups, * means the statistical difference between the LA and CTRL groups. **(E)** Relative wound areas on Day 9 (Fold of the wound on Day 3) were analyzed (n = 6). **(F)** Skin wound hematoxylin and eosin (HE) staining on Day 5. Scale bar = 400 µm. **(G)** Skin wound HE staining on Day 12. Data represents mean ± SEM, *p < 0.5, **p < 0.01, ##*p* < 0.01.

Our previous study showed that pro-inflammatory macrophages, following neutrophils, started to dominate on Day 3 post-injury and subsequently began to transition to a reparative phenotype 10. To avoid possible influence on neutrophils, we designed the drug-administration protocol as shown in Figure S1A. We topically administered exogenous lactate (20mM) on Days 3 and 5 post-injury. As data showed, the number of pro-inflammatory macrophages (iNOS+ CD68+ positive cells) in the wound tissues of the LA group was significantly lower, while the level of reparative macrophages (ARG1+ CD68+ positive cells) was higher than that of the CTRL group on Day 5 post-injury. (Figures 4A, B; Figures S1B, C). Compared with the LA group, in the LA+Pi group, the number of pro-inflammatory macrophages was higher (Figure 4A), while reparative macrophages were lower (Figure 4B).

The photographs of the healing wounds show that the wound-healing speed of mice in the LA group was significantly faster than in the CTRL group (Figures 4C, D; Figures S1D, E), while the wound-healing speed of the LA+Pi group was markedly slower than that of the LA group (Figures 4C, D). As the data shows, the wound area in the LA group on Day 9 was significantly smaller than that in the CTRL group (Figure 4E; Figure S1F), while the wound area in ​​the LA+Pi group on Day 9 was considerably more extensive than that in the LA group (Figure 4E).

In addition, the speed of wound-healing was verified by re-epithelialization on Day 5 post-injury. The length of the migrating epithelial tongue in the LA group was markedly longer than that in the CTRL group (Figure 4F; Figure S1G), while the length in the LA+Pi group was considerably shorter than that in the LA group (Figure 4F). Furthermore, there was no significant difference in skin quality among the three groups on Day 12 post-injury (Figure 4G; Figure S1H). This indicates that lactate intervention did not reduce the healing quality of the wound. Taken together, these results show that, in mice, topically administering exogenous lactate promotes the transition of wound macrophages from a pro-inflammatory phenotype to a reparative phenotype and accelerates wound healing by promoting PKM2 pyruvate kinase activity.

### Lactate promotes K62 lactylation of PKM2

The above results suggest that lactate activates PKM2. Therefore, we further explored the underlying mechanism. Numerous studies have shown that PTMs play a central role in the functional regulation of PKM2. As a potential modification substrate, lactate has recently been reported to modify proteins directly 9. It remains unknown whether lactylation modification of PKM2 exists and how lactylation modification modulates the function of PKM2.

**Figure 5:**
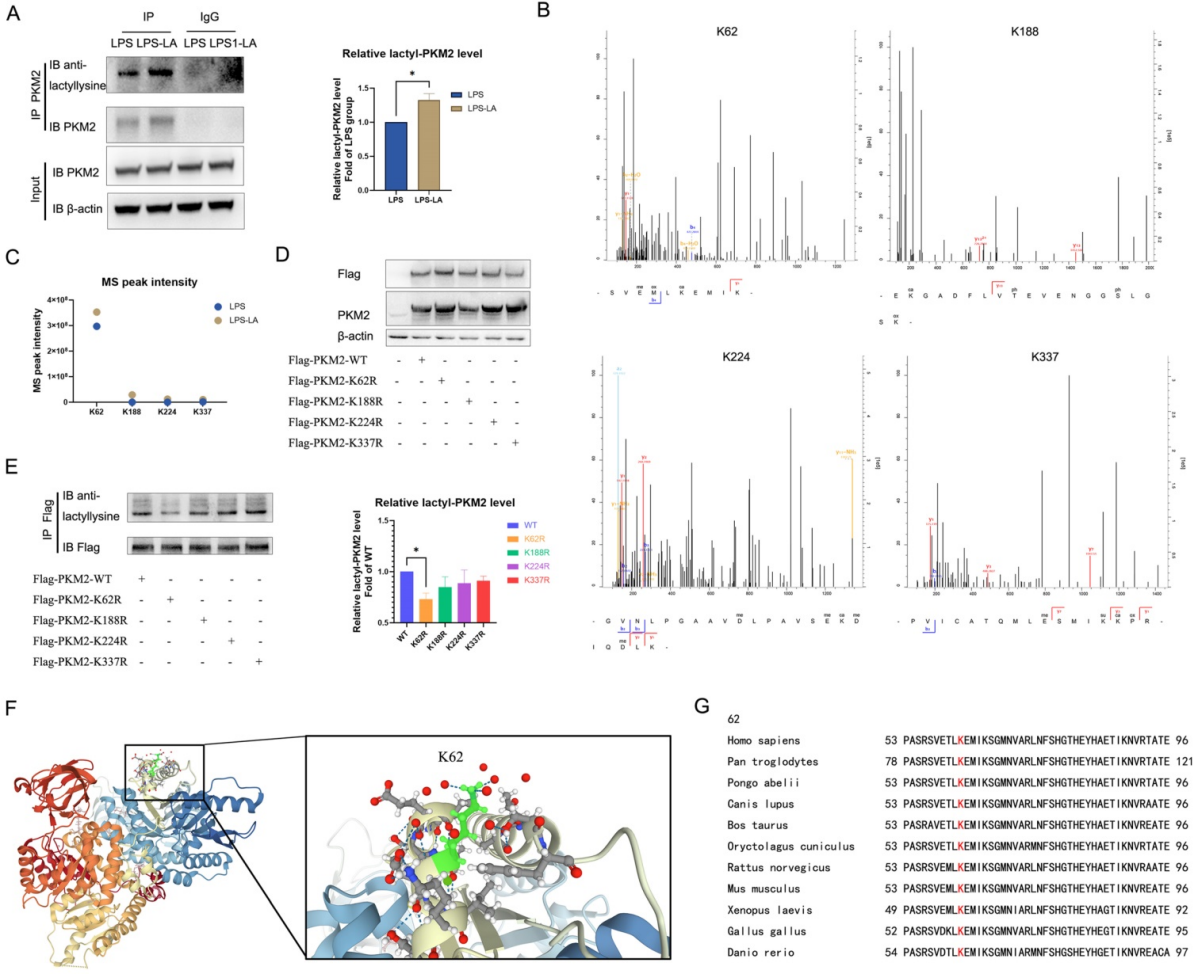
** Lactate promotes K62 lactylation of PKM2. (A)** Naïve bone-marrow-derived macrophage cells were incubated with 100 ng/ml lipopolysaccharide (LPS) for 4 hr, followed by vehicle (LPS) or 20 mM lactate (LPS+LA) for 20 hr. Then cell proteins were pulled down by PKM2 antibody and detected with anti-lactyllysine antibody. **(B)** We detected possible lactylation sites of PKM2 in LPS-induced BMDM cells through immunoprecipitation (IP)-mass spectrometry analysis. The four possible lactylation sites of PKM2 through IP-mass spectrometry are shown. **(C)** The observed mass spectrum peak intensity of lactyl-lysine-containing peptides in BMDM cells (incubated with 20 mM lactate or not). **(D)** K62R, K188R, K224R, and K337R site mutations, or wild-type flag-PKM2 overexpressed 293T cells were constructed respectively through overexpression plasmid. Cell proteins were analyzed by western blotting (WB) for PKM2 levels. **(E)** Flag-PKM2 overexpressed 293T cells proteins were pulled down by flag antibody and detected with anti-lactyllysine antibody. **(F)** Ribbon diagram of the crystal structure of human PKM2 protein (PDB entry 6B6U). **(G)** The K62 site in PKM2 is conserved. The sequences around PKM2 K62 from different species were aligned. Conserved lysine residues corresponding to human PKM2 K62 are marked in red. WB data represents three independent experiments. Data represents mean ± SEM. *p < 0.05.

In LPS-induced BMDM cells, we used the PKM2 antibody to pull down PKM2 and use the anti-lactyllysine antibody to detect the level of PKM2 lactylation. We observed that PKM2 was modified by lactylation, which was significantly enhanced after treatment with 20 mM lactate for 24 hr (Figure 5A). Next, we detected possible lactylation sites of PKM2 in LPS-induced and LPS+LA-induced BMDM cells through IP-mass spectrometry analysis. The potential lactylation sites of PKM2 are shown in Table S1.

**Figure 6:**
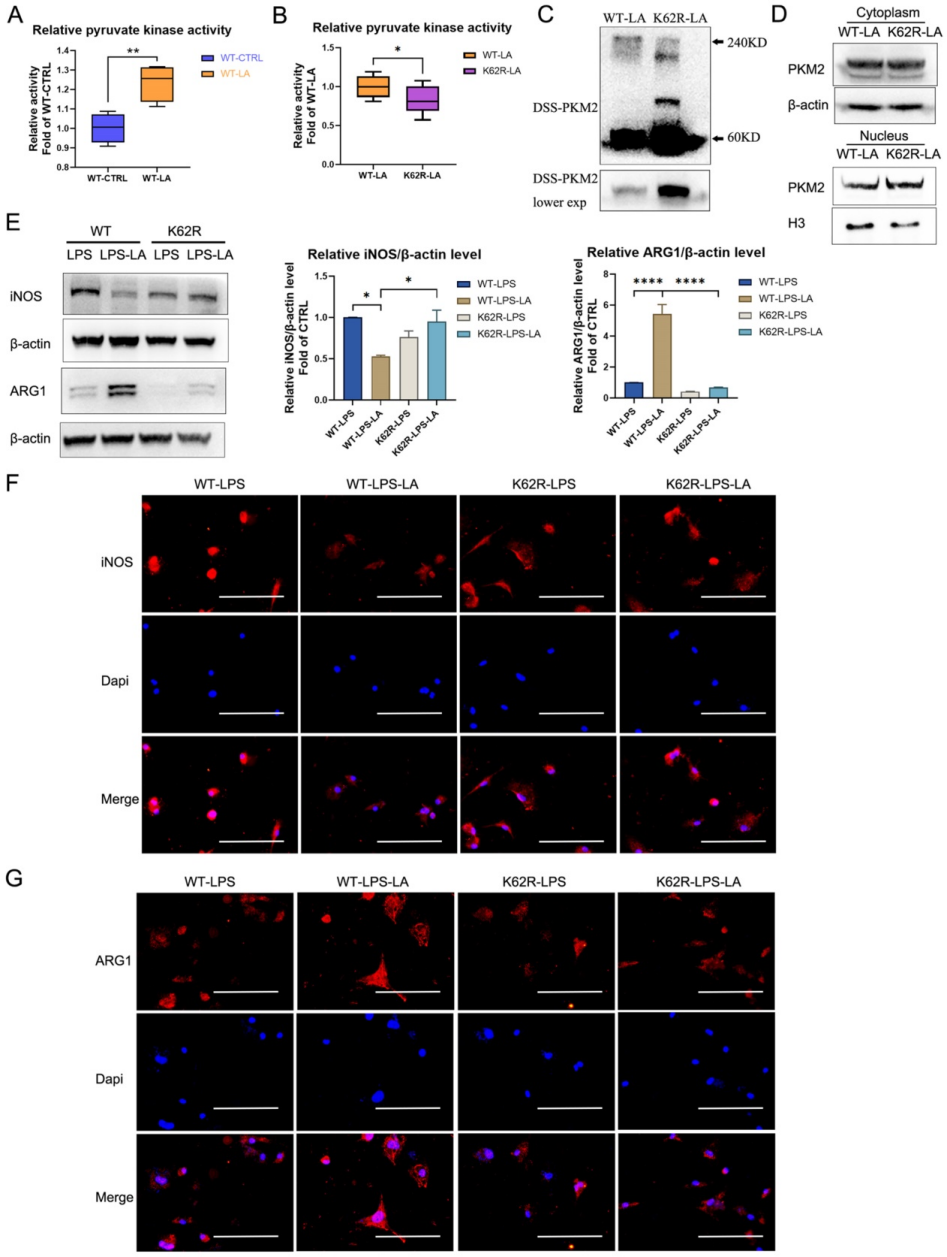
** The K62R mutant reverses the regulation of lactate on PKM2 pyruvate kinase activity and macrophage phenotype transition. (A)** Wild-type flag-PKM2 overexpressed 293T cells were incubated with vehicle (WT-CTRL) or 20 mM lactate (WT-LA) for 24 hr. Then cell lysates were assayed for pyruvate kinase activity (n = 4). **(B)** WT (WT-LA) or K62R site mutation (K62R-LA) flag-PKM2 overexpressed 293T cells were incubated with 20 mM lactate for 24 hr. Then cell lysates were assayed for pyruvate kinase activity (n = 8). **(C)** 293T cells were collected and crosslinked with disuccinimidyl suberate (DSS). The tetrameric (240KD), dimeric (120KD), and monomeric (60KD) forms of PKM2 were analyzed by western blotting (WB). **(D)** Cytosolic and nuclear proteins were purified from 293T cells separately for PKM2 level analysis. WB data represents three independent experiments. **(E)** WT or K62R site mutation PKM2 overexpressed BMDM cells were constructed through the lentiviral vector. Then the PKM2-overexpressed BMDM cells were incubated with 100 ng/ml LPS ± 20 mM lactate for 24 hr. Cell proteins were analyzed by WB for the iNOS and ARG1 levels. The blots are representative of three independent experiments. Immunofluorescence staining of iNOS **(F)** or ARG1 **(G)** was performed. Data represents mean ± SEM. Scale bar = 100 µm. *p < 0.5, **p < 0.01, ***p < 0.001.

We noted four sites (K62, K188, K224, and K337) with the most significant increase in MS peak intensity after the intervention of lactate (Figures 5B, C), which suggested that these sites were more sensitive to the lactate incubation. Then, by ectopically overexpressing flag-tagged PKM2 into 293T cells through overexpression plasmid, we constructed K62R, K188R, K224R, and K337R site mutations or WT PKM2 overexpression 293T cells. As shown in Figure 6D, compared with the control group, the level of PKM2 in PKM2-overexpressed cells was significantly up-regulated.

Next, we used a flag antibody to pull down PKM2 to detect lactylation levels. The results confirm that the K62R site mutation significantly reduced PKM2 lactylation, while the other three site mutations had no significant effect on the lactylation level (Figure 5E). These results suggest that the K62 site is the major lactylation site of PKM2. The tertiary structure of PKM2 showed that K62 is located in the A domain of the PKM2 protein (Figure 5F) and is adjacent to a critical, active site, S362. As shown in Figure 5G, K62 is conserved in species ranging from Danio rerio to various mammals. These facts support the hypothesis that the K62 site is a potential regulatory site for the function of PKM2.

### The K62R mutant reverses the regulation of lactate on PKM2 pyruvate kinase activity and macrophage phenotype transition

We further evaluated the regulative effect of lactylation at the K62 site on PKM2 function. As shown in Figure 6A, lactate incubation markedly promotes PKM2 enzyme activity in WT PKM2 overexpression 293T cells. The K62R mutation significantly inhibits PKM2 enzyme activity in 293T cells following lactate incubation (Figure 6B; K62R-LA group vs WT-LA group). The DSS crosslinking of lysates from lactate-incubated 293T cells suggests that the level of the PKM2 tetrameric form (240KD) in the K62R-LA group was significantly lower than that in the WT-LA group (Figure 6C). In addition, the level of PKM2 in the nuclei of the K62R-LA group was approximately 1.41 times higher than that in the WT-LA group in lactate-incubated 293T cells (Figure 6D). These results confirm that the K62 site has a marked effect on the function of PKM2 and demonstrate that lactate partially promotes PKM2 enzyme activity through lactation of the K62 site.

Next, we overexpressed the K62R mutation or WT PKM2 in BMDM cells using the lentiviral vector. After LPS incubation for 48 hr, lactate increased the ARG1 level but decreased the iNOS level in WT BMDM cells (Figures 6E-G; WT-LPS-LA group vs WT-LPS group). This effect of lactate was significantly weakened in the K62R-mutation BMDM cells (Figures 6E-G; K62R-LPS-LA group vs K62R-LPS group). Compared to the WT-LPS-LA group, the K62R-LPS-LA group showed a higher iNOS level and a lower ARG1 level (Figures 6E-G). These results support the conclusion that lactate partially promotes the transition of LPS-induced macrophages to a reparative phenotype by the lactylation of PKM2 at the K62 site.

## Discussion

PTMs play a crucial role in the regulation of PKM2 23, 28. They significantly alter the structural and functional properties of PKM2, such as catalytic activity and subcellular localization. As a potential modification substrate, lactate has recently been reported to directly modify proteins, which alter the structure and function of proteins, such as histone 9, HMGB1 37, and METTL3 38. These studies have dramatically contributed to our understanding of the non-metabolic functions of lactate in physiology and disease. However, there remains considerable doubt about whether lactylation in PKM2 exists and how lactylation modulates the function of PKM2. To the best of our knowledge, this study is the first to document that PKM2 can be modified by lactylation, which can be promoted by exogenous lactate. Moreover, we identified multiple lactylation sites in PKM2 and defined K62 as the potential lactylation target. We proved that an increase in lactylation of PKM2 at K62 inhibits its tetramer-to-dimer transition, promoting its pyruvate kinase activity and reducing nuclear distribution. Our study enriches knowledge about the endogenous regulative mechanism of PKM2 and enhances understanding of the physiological significance of lactylation modification.

The present study suggests that an increase in lactylation of PKM2 at the K62 could directly forge PKM2 into a more active tetrameric form (Figure 6C) and promote its pyruvate kinase activity (Figures 6A, B). These data reveal that lactylation may be an essential mechanism for controlling the PKM2 pyruvate kinase activity, and the K62 site might be an important allosteric site of PKM2. However, it remains unclear how lactylation at the K62 site regulates PKM2 conformation. Previous studies have stated that the active site, fructose-1,6-bisphosphate (FBP)-binding pocket, and the amino acid binding pocket regulate PKM2 conformation and pyruvate kinase activity 39-43. Binding to FBP at the FBP-binding pocket forges PKM2 into a more active tetramer. In addition, the binding of serine to the amino acid binding pocket causes the allosteric pocket to shrink. As a result, serine aids in stabilizing the tetrameric conformation of PKM2 42. In contrast, cysteine binding to PKM2 at the amino acid binding pocket could perturb the dynamics of polar amino acids in the amino acid binding pocket, inhibiting PKM2 pyruvate kinase activity by destabilizing the tetrameric conformation of PKM2 39. Moreover, allosteric inputs from distinct effectors show functional cross-talk to control the activity of PKM2 40. For example, in the presence of oxalate, PKM2 adopts an active tetrameric conformation which is more amenable to binding serine rather than cysteine 39. Meantime, the cysteine-mediated PKM2 inhibition could be reversed by FBP 39. The relative concentrations of allosteric effectors can fine-tune PKM2 pyruvate kinase activity through competition at allosteric sites between activators and inhibitors. As K62 locates in the A domain, which is adjacent to the active site and amino acid binding pocket but distant to the FBP-binding pocket of PKM2, we speculated that the K62 lactylation might spatially aid in shrinking the amino acid binding pocket or alter the binding affinities of distinct allosteric effectors for PKM2, which is more amenable to binding activators than inhibitors, thereby ultimately stabilizing the tetrameric conformation and promoting its pyruvate kinase activity. The detailed mechanism of K62 site lactylation in regulating the conformation of PKM2 remains to be further elucidated.

PKM2 is expressed both in cancer cells and in normally proliferating cells, such as immune cells 44, 45. Previous studies have confirmed that PKM2 is the dominant PK subtype in CD4+ T cells, RAW264.7 cells, and microglia 46, 47. By detecting the expression of PKM2 and PKM1 using RT-qPCR, our study shows that PKM2 is more abundantly expressed than PKM1 in BMDM cells. Moreover, as shown in Figure S2, we detect the protein levels of PK subtypes' expression in different cells. Previous studies showed that PKM1 was primarily found in the myocardium, and PKM2 was mainly expressed in 293T cells 45, 48. The data in Figure S2 confirms these findings. Meanwhile, the PKM2 level is dramatically higher than the PKM1 level in naive BMDM cells. These findings further confirm that PKM2 is more abundantly expressed than PKM1 in BMDM cells.

PKM2 is a crucial regulator of immunometabolism 49. Other researchers have observed that promoting PKM2 pyruvate kinase activity inhibits the Warburg effect and lactate production, a phenomenon known as the PKM2 paradox in the Warburg effect 21, 25. A study has reported that promoting PKM2 pyruvate kinase activity using the well-characterized small molecules DASA-58 and TEPP-46 counteracts the excessive rate of glycolysis induced by LPS. This could attenuate an LPS-induced pro-inflammatory M1 macrophage phenotype 21. Promoting PKM2 pyruvate kinase activity reduces the Warburg effect by promoting oxaloacetate accumulation 25. In addition, promoting PKM2 pyruvate kinase activity boosts IL-10 levels 50, inhibiting glycolysis by downregulating glycolytic genes and preventing GLUT1 translocation from intracellular vesicles to the plasma membrane 51. In contrast, the monomeric and dimeric forms of PKM2 translocate to the nucleus, enabling the expression of numerous glycolytic enzymes by interacting with Hif-1α 21 and upregulating the expression of c-Myc 26. Another study has demonstrated that PKM2 enzymatic inhibitor compound 3K significantly blocks Anx A5-attenuated M1 macrophage glycolysis, promoting M1-related factors 52. Consistent with these findings, our study shows that compound 3K can increase glycolysis levels. Moreover, the role of PKM2 in regulating glycolysis was also confirmed in CD4+ T cells in another study 47. However, another study suggests that a different PKM2 enzymatic inhibitor, shikonin, which has weaker PKM2 inhibitory activity than compound 3K but similar selectivity 53, inhibited LPS-induced glycolysis in macrophages 54. The mechanisms behind the different effects of compound 3K and shikonin on glycolysis in macrophages, however, are unclear. It has been reported that shikonin may act in a PKM2-independent manner 49. Thus, in contrast to compound 3K, shikonin might have other mechanisms targeting macrophage glycolysis.

Although PKM2 directs lactate production, it remains largely unknown whether lactate regulates PKM2 pyruvate kinase activity or function. In this study, we found, for the first time, that lactate activates PKM2 in a feedback loop and further elucidated that the effect was partially produced by promoting PKM2 lactylation. Given the role of PKM2 in regulating glycolysis, lactate theoretically can inhibit glycolysis by activating PKM2. As expected, a recent study has shown that long-term treatment with lactate inhibits glycolysis in pro-inflammatory macrophages 17. However, the underlying mechanism is unclear. We confirmed the glycolysis inhibiting role of lactate in LPS-induced macrophages and further suggested that lactate inhibits glycolysis in part by activating PKM2. These findings may deepen the understanding of how lactate regulates glycolysis. The fact that lactate activates PKM2 in a feedback loop helps to suppress the excessive Warburg effect in a lactate-enriched environment, which may restrict the exaggerated inflammatory response in pro-inflammatory macrophages.

Macrophages naturally transition from a pro-inflammatory state to a reparative state, which is crucial for natural wound-healing 7, 10. This reparative macrophage transition can occur sequentially after the remove of inflammatory stimuli, without exogenous signals 3. However, whether the increase in reparative-state macrophages originated from differentiation from pro-inflammatory macrophages remains inconclusive. Arginine metabolism is central to the diverse metabolic fates of inflammatory macrophages. iNOS, which promotes nitric oxide (NO) formation from arginine and is necessary for inflammation and microbe killing, is expressed in pro-inflammatory macrophages. ARG1, which limits arginine availability for NO production and is essential for tissue regeneration, is expressed in reparative macrophages. A recent study reports a time-dependent change in macrophage phenotype after LPS incubation, during which there was decreased expression of iNOS and increased ARG1 9. This indicates that a macrophage cell can participate sequentially in both the induction and the resolution of inflammation. However, the molecular mechanism underlying this event remains largely unknown. In recent years, insights into the critical role of metabolic adaptation in macrophage phenotype have emerged. 21 Studies have suggested that inhibition of glycolysis by 2-DG modulates macrophage polarization, increasing levels of ARG1 and decreasing expression of iNOS 19, 55. In addition, PKM2 is an essential molecular determinant of the Warburg effect and bridges metabolic and inflammatory functions 33, 49. Inhibiting PKM2 promotes macrophage glycolysis and inflammatory responses 56, while activating PKM2 has the opposite effect 57. These facts suggest that metabolic adaptation is an essential determinant of the phenotype of macrophages.

Recent studies found that long-term lactate exposure reduced iNOS 36 and glycolysis inhibition 17 in pro-inflammatory macrophages. We confirmed this phenomenon and further suggested that inhibiting PKM2 reverses the effect of lactate on glycolysis inhibition and iNOS reduction. These facts indicate lactate may inhibit glycolysis and iNOS levels by activating PKM2. In addition, a recent study reports that intervention with lactate increased ARG1 levels in LPS-induced macrophages 9. However, a subsequent study found that intervention with lactate in naïve macrophages did not induce ARG1 upregulation 58. These results suggest that additional LPS signaling is needed for lactate to regulate ARG1 in macrophages. Consistent with previous findings 21, our study found that LPS significantly increases PKM2 expression in macrophages. Promoting PKM2 pyruvate kinase activity in LPS-induced macrophages inhibits glycolysis and promotes ARG1 expression. Furthermore, we demonstrated that lactate inhibits glycolysis and promotes ARG1 expression by activating PKM2. This partially explains why lactate cannot promote ARG1 in naïve macrophages, whereas it can promote ARG1 in LPS-induced macrophages. In short, our study suggests that the metabolic reprogramming regulated by PKM2 may be an important reason for the phenotypic transition of macrophages that is induced by lactate.

Although the wound healing process has evolved over thousands of years, and both the molecular players and timings in the wound healing process seem to have been selected through evolution as optimal, however, the existing protocol of wound healing is based on a balance of infection clearance and tissue regeneration in the natural environment. First, the wound undergoes an inflammatory response to clear pathogens, which causes damage to the surrounded tissues. Pro-inflammatory macrophages are essential for microbe killing but unfavorable for tissue regeneration 5; reparative macrophages are primarily responsible for wound healing 8. Thus, macrophages play a vital role in the wound-healing process. It has been noted that injuries to the mucosal surface often heal faster than cutaneous wounds 59, and many studies have attempted to stimulate macrophages toward a reparative phenotype for better wound healing outcomes 12, 13. These facts indicate that the healing process of cutaneous wounds may not be optimal, and promoting macrophage transition to accelerate healing is mechanistically feasible. Other studies have suggested that lactate can accelerates the transition of macrophages to the reparative phenotype and, therefore, wound healing 60. In this study, we confirmed these effects of lactate and further confirmed that lactate may partially function by activating PKM2. In patients with large skin defects, such as severe burns, it is beneficial for the speed of recovery if wound healing can be accelerated. In addition, since there is an increased chance of hypertrophic scarring if a wound requires more than 2 weeks to re-epithelialize 61, it is essential to accelerate wound healing to avoid scarring.

In summary, our study identifies PKM2 as a lactylation substrate for the first time. We confirm that lactylation increases the pyruvate kinase activity of PKM2, reducing its tetramer-to-dimer transition and nuclear distribution. We have also found that lactate causes decreased glycolysis by activating PKM2, promoting the transition of pro-inflammatory macrophages to a reparative phenotype.

## Supplementary Material

Supplementary figures.

Supplementary tables.

## Figures and Tables

**Table 1:** Sequences of primers used in real-time quantitative polymerase chain reaction

Gene	Primer sequences (5′-3′)	
PKM1 (Mouse)	Forward	CTATCCTCTGGAGGCTGTGC
	Reverse	CCATGAGGTCTGTGGAGTGA
PKM2 (Mouse)	Forward	CGCCTGGACATTGACTCTG
	Reverse	GAAATTCAGCCGAGCCACATT
β-actin (Mouse)	Forward	GTGACGTTGACATCCGTAAAGA
	Reverse	GCCGGACTCATCGTACTCC
