# Supplementary material for: Lactylation of PKM2 Suppresses Inflammatory Metabolic Adaptation in Pro-inflammatory Macrophages
Source: Int J Biol Sci. 2022 Oct 24;18(16):6210–25. doi: 10.7150/ijbs.75434 (PMC9682528; doi:10.7150/ijbs.75434)
Supplement: Supplementary file 1 — Supplementary figures. [file ijbsv18p6210s1.pdf]

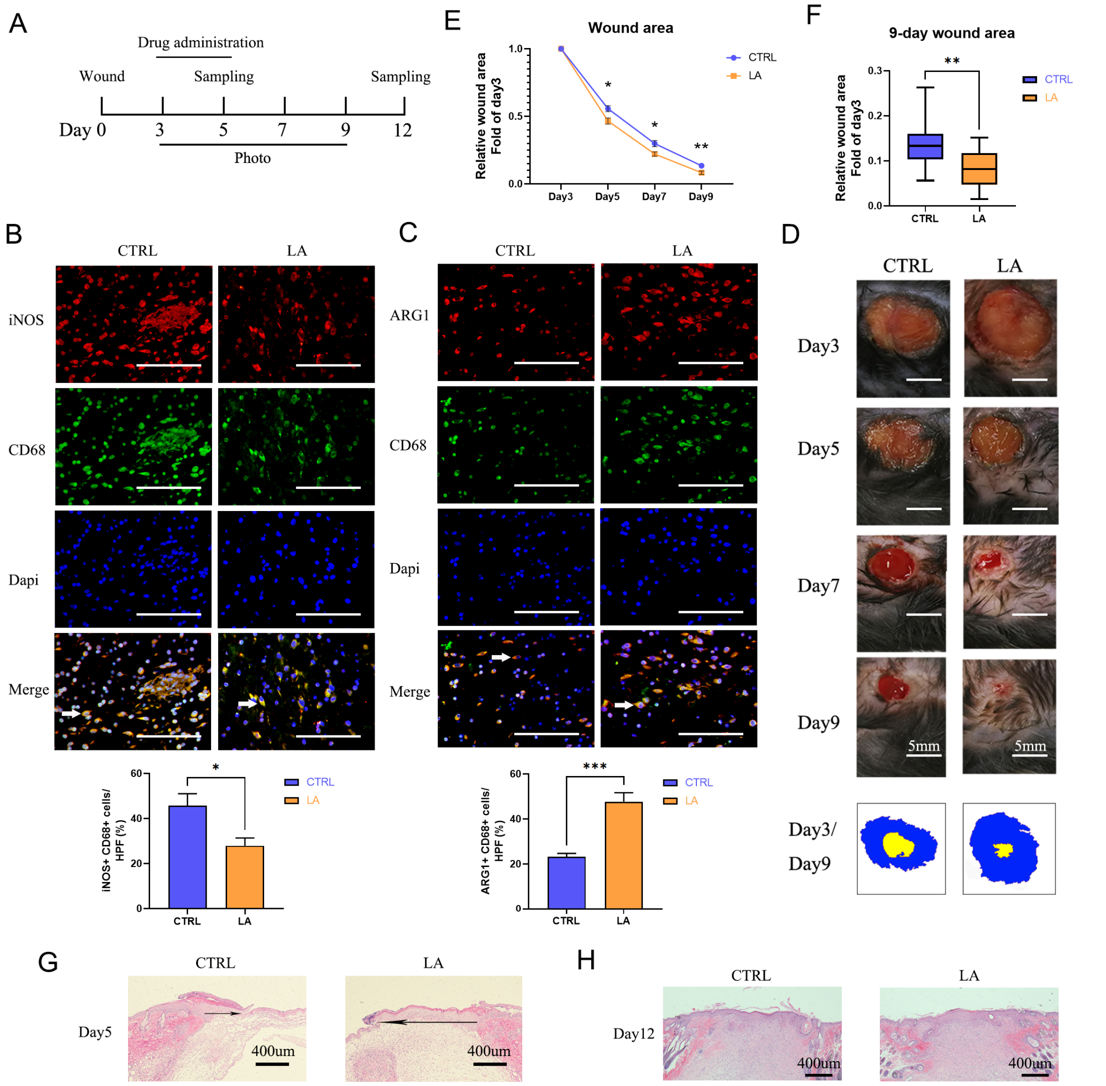

Figure S1 Lactate promotes the transition of wound macrophages to a reparative phenotype and accelerates wound healing in mice

Twenty mice were randomly divided into two intervention groups: the CTRL group (treated with vehicle, n=10) and the LA group (treated with 20 mM lactate, n=10). **(A)** The protocol of in vivo study was shown. Immunofluorescence staining with iNOS + CD68 **(B)** or ARG1 + CD68 **(C)** was performed to analyze wound macrophages on Day 5 post-injury. Scale bar = 100 μm. n = 6. **(D)** The photographs of skin wounds and schematic diagram of comparison of wound area on Day 3 and Day 9. Scale bar = 5 mm. **(E)** Statistical results of relative wound area (fold of the

wound on Day 3). **(F)** Relative wound area on Day 9 post-injury (fold of the wound on Day 3) was analyzed (n = 14). **(G)** Skin wound hematoxylin and eosin (HE) staining on Day 5. Scale bar = 400 μm. **(H)** Skin wound HE staining on Day 12. Data represents mean ± SEM, *$p < 0.5$, **$p < 0.01$.

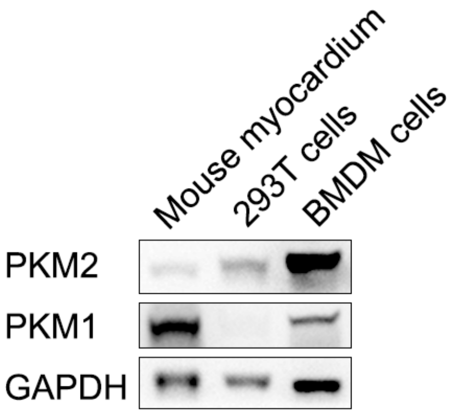

Figure S2 PKM2 is far more expressed than PKM1 in BMDM cells

The protein levels related to the expression of PKM1 and PKM2 in mouse myocardium, 293T cells, and mouse bone-marrow-derived macrophage cells were detected.
